# From Genome to Pharmacome: Current Status and Future Perspectives of Multi-Omics Integration in Traditional Chinese Medicine Research

**DOI:** 10.3390/genes17060634

**Published:** 2026-05-30

**Authors:** Tengfei Yu, Changting Chen, Peng Hu, Yunlian Zou, Jinping Zhang, Qianze Zhu, Tonghua Yang

**Affiliations:** 1 Faculty of Life Science and Technology, Kunming University of Science and Technology, Kunming 650500, China; 20201136011@stu.kust.edu.cn (T.Y.); cct11231231@163.com (C.C.); hupengkkxx@126.com (P.H.); 2 Medical School, Kunming University of Science and Technology, Kunming 650500, China; 3 Department of Integrated Chinese and Western Medicine, The First People’s Hospital of Yunnan Province, Kunming 650032, China; 4 Yunnan Provincial Clinical Medical Center for Blood Diseases and Thrombosis Prevention and Treatment, The First People’s Hospital of Yunnan Province, Kunming 650032, China; zouyunlian825046@sina.com (Y.Z.); 300889@ynpfh.cn (J.Z.); 5 Department of Hematology, The First People’s Hospital of Yunnan Province, Kunming 650032, China

**Keywords:** multi-omics, high-throughput sequencing, traditional Chinese medicine, systems biology, mechanism of action

## Abstract

High-throughput sequencing and multi-omics are transforming Traditional Chinese Medicine (TCM) research from empirical descriptions toward data-driven mechanistic analyses. Unlike earlier systems pharmacology frameworks that relied primarily on static network topology and docking-based target prediction, current multi-omics approaches integrate genomic, transcriptomic, proteomic, and metabolomic data to capture dynamic, multi-scale biological responses. This review summarizes recent progress in four related areas: (i) genomic and epigenomic dissection of geo-authentic (Daodi) medicinal materials; (ii) biosynthetic pathway elucidation for major bioactive compound classes; (iii) synthetic biology platforms for heterologous production; and (iv) systems pharmacology integration for mechanism-of-action studies. We identify a central, recurrent gap: most published multi-omics analyses remain at the level of statistical association, and the biosynthetic and pharmacological pathways inferred from such data have not been validated at the causal level. To address this, we propose a tiered experimental validation framework—from biochemical target engagement through genetic perturbation to in vivo functional confirmation—and an iterative computational–experimental feedback loop. We further outline practical priorities for future work, including standardized data formats, community-endorsed metadata checklists, and coordinated DBTL pilot projects. By connecting descriptive multi-omics patterns to experimentally testable mechanistic models, TCM research can move toward precision-oriented medicine while preserving the multi-component character of traditional formulations.

## 1. Introduction

TCM has clear clinical value in the intervention and control of chronic diseases. Its core advantage lies in the composition diversity and multi-target regulatory mechanism. Such pharmacological features cannot be well interpreted by the classic single-target research model. This complexity does not fit easily into conventional single-target frameworks [1].

High-throughput sequencing has reshaped the landscape of TCM research. The field is no longer limited to empirical observation and has stepped into systematic analytical research. Herb genomics occupies a core position in this research transformation.

Genome data of common medicinal plants lay a foundation for exploring the biosynthesis of secondary metabolites. Representative species include *Salvia miltiorrhiza* [2,3] and *Panax notoginseng* [4,5]. Relying on existing genomic resources, multi-omics technology supports a finer dissection of synthetic pathways. It can achieve accurate resolution at the tissue and cell levels [6,7].

Multi-omics technologies can trace the source of active ingredients in TCM. It remains difficult to clarify their specific pharmacological mechanisms. Network pharmacology is widely adopted to map relationships among compounds, targets, and signaling pathways. Relevant foundational studies have supported this analytical system [1,8]. Machine learning has gradually become an effective tool to boost prediction accuracy [9]. Computational prediction has outpaced experimental verification: most proposed mechanistic explanations remain based on correlation rather than causal evidence, a gap that recurs throughout the field and is addressed in detail in Section 5.4.

There is no unified research system to integrate medicinal plant genetics, biosynthesis, and pharmacological networks. Cross-omics analysis inevitably faces data processing obstacles and heterogeneous differences. These bottlenecks must be addressed first to construct an integrated research system. It is also essential to accumulate sufficient functional validation evidence. Such empirical data can support a mechanistic interpretation, rather than merely relying on correlation analysis.

A systematic literature search was conducted in PubMed, Web of Science, and Scopus for articles published between January 2010 and March 2026. The search combined terms related to multi-omics (“genomics”, “transcriptomics”, “proteomics”, “metabolomics”, “multi-omics”, “high-throughput sequencing”), traditional Chinese medicine (“TCM”, “Chinese herbal medicine”, “medicinal plant”), and methodological approaches (“network pharmacology”, “systems biology”, “biosynthetic pathway”, “synthetic biology”). Only peer-reviewed English-language articles were included. Preprints were not considered. Reference lists of key reviews were manually screened to identify additional relevant studies. In total, 114 articles were selected for inclusion based on relevance to the review scope.

**Figure 1:**
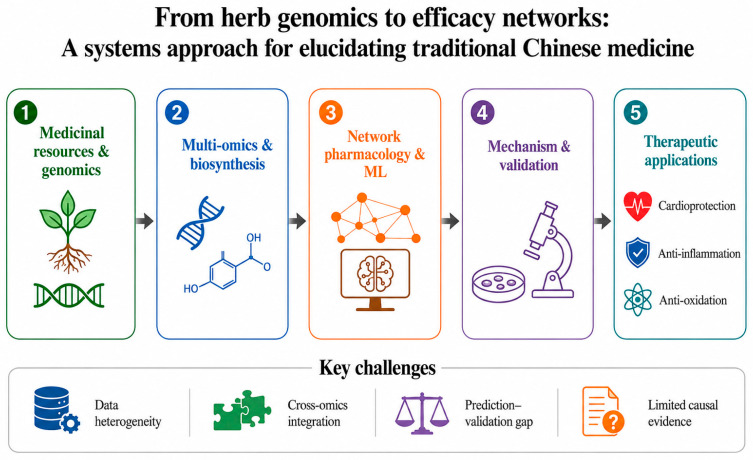
A systematic research framework connecting medicinal resource characterization with therapeutic applications. Genomic analysis of medicinal plants reveals the intrinsic basis of secondary metabolite biosynthesis. Multi-omics technologies—transcriptomics, proteomics, metabolomics, spatial transcriptomics, and single-cell sequencing—jointly support biosynthetic pathway reconstruction. These datasets inform network pharmacology and machine learning models that map relationships among active components, targets, and signaling pathways. Subsequent experimental work validates predicted mechanisms in chronic disease settings. Abbreviations: ML, machine learning.

This paper summarizes recent progress in genome sequencing and multi-omics research. We also discuss how these technologies can clarify the biological complexity behind TCM research (Figure 1). Compared with earlier systems pharmacology frameworks that mainly relied on static network topology and docking-based target prediction, current multi-omics approaches enable dynamic, data-driven integration across transcriptional, proteomic, and metabolic layers—an advance that both expands analytical scope and raises new challenges in standardization and validation.

## 2. Unraveling Geo-Authenticity: Environmental Factors and Genomic Underpinnings

TCM geo-authenticity, also referred to as Daodi, refers to medicinal plant materials grown in designated geographical areas. Such medicinal herbs show better clinical efficacy. They also maintain stable chemical composition characteristics compared with ordinary varieties. Climate and soil conditions are core environmental factors. They have long been regarded as the main causes of TCM geo-authentic traits, yet the molecular and genetic basis behind the formation of geo-authentic quality cannot be clarified.

Recent multi-omics studies have emphasized the role of genotype–environment interactions. These interactions are crucial for the formation of chemical diversity in medicinal plants. Comparative genomic analyses have found potential associations. Specifically, the expansion of secondary metabolism-related gene families is linked to the diversification of bioactive compounds [10]. At the population level, there are genetic differences among different geographical regions. These differences are consistent with the variation of key biosynthetic pathways. This correlation lays a foundation for the regional differences in metabolite composition [11]. At the transcriptional level, enzyme expression varies across different tissues or environments. This differential expression is closely related to the accumulation of characteristic metabolites [12].

A well-documented example linking genotype, metabolite profile, and therapeutic outcome comes from *Panax ginseng*. Liu et al. [11] performed population genomic analysis of ginseng accessions using high-density genic SNP markers, identifying genetic clusters corresponding to geographic origins and cultivar types. These genetic groupings were significantly associated with ginsenoside composition, and the observed chemotypic variation may contribute to differences in pharmacological potential. Similarly, in *S. miltiorrhiza*, substantial variation in tanshinone content has been reported among cultivars, and genomic analyses have identified candidate variants associated with these chemotypic differences [2,3]. These examples illustrate the feasibility and practical value of linking genomic variation to therapeutically relevant chemical phenotypes—a connection that remains underexploited for most medicinal species.

Environmental factors can regulate secondary metabolism in medicinal plants. Typical examples include drought and temperature fluctuations. These factors function by activating stress response pathways and changing carbon distribution and energy utilization patterns [13]. Taking nitrogen metabolism as an example, genetic differences in this process affect the balance between biomass accumulation and specialized metabolite synthesis. This balance directly influences the medicinal quality of plants [14].

High-resolution reference genomes and comparative datasets are now more accessible. This accessibility has changed the understanding of TCM geo-authenticity. It is no longer regarded as a trait determined by a single factor, but a process regulated by multiple factors [15]. Multi-omics integration technology has gradually matured. It has now become a practical tool for origin traceability and quality evaluation of medicinal materials.

**Figure 2:**
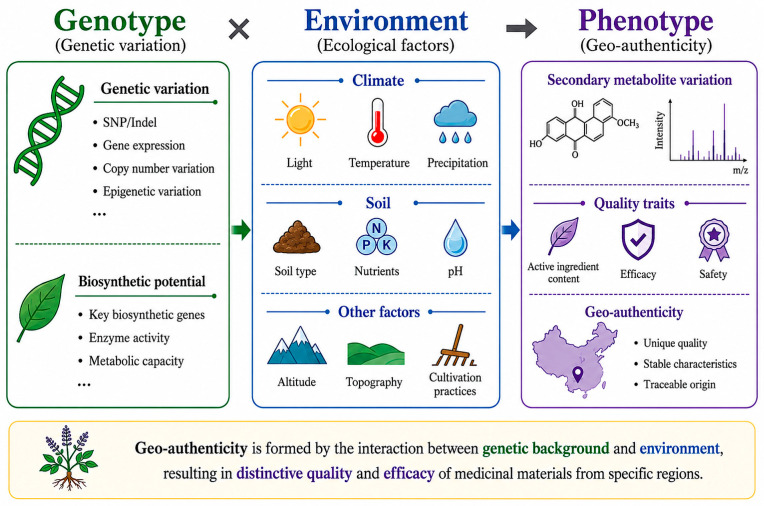
Genotype–environment interactions regulate geo-authentic medicinal materials. Genetic variation (sequence polymorphisms, gene expression, biosynthetic capacity) and environmental factors (microclimate, soil, cultivation) interact to influence secondary metabolite synthesis and active compound composition, producing metabolite profiles closely associated with the efficacy and safety of geo-authentic medicinal plants. The colors indicate different conceptual categories: green represents genotype/genetic background, blue represents environmental factors, and purple represents phenotype/geo-authenticity-related traits. Abbreviations: SNP, single-nucleotide polymorphism; Indel, insertion/deletion; N, nitrogen; P, phosphorus; K, potassium; m/z, mass-to-charge ratio.

Current research faces a key challenge: to directly link genomic variation with metabolite profiles and clinical efficacy across different biological scales (Figure 2).

### 2.1. Genotype × Environment: Genetic Architecture Underlying Daodi Quality

Genotype-by-environment (G × E) interactions shape geo-authentic medicinal materials. Environmental factors alone cannot explain their formation. High-quality reference genomes are now easier to obtain. Expanded gene families contribute to the diversification of bioactive compounds [10]. These metabolic pathways are sensitive to environmental shifts. Gene expression shifts in response to environmental change. This transcriptional plasticity reflects G × E interactions. Metabolic outcomes also depend on the environmental context [16].

Long-term environmental selection drives local adaptation. Stable genetic differences arise among populations from different regions. *Forsythia suspensa* offers an example. Population genomic analyses have detected adaptive divergence across its ecological niches [17,18]. These kinds of changes do not come from single genes alone. Instead, they involve coordinated effects across multiple genes, which is what we call polygenic adaptation [19]. Those genome-wide patterns serve as the molecular foundation behind stable, region-specific quality traits seen in geo-authentic TCM herbs. In short, geo-authenticity means a heritable and repeatable phenotype—specific genotypes produce it when grown under defined environmental conditions. However, a clear gap remains that has not been closed. Trying to link genetic variation with metabolic phenotypes and then to pharmacological effects remains a tough challenge. If one only looks at single omics layers or just one biological scale, one simply cannot capture the full complexity of the system.

These findings should be interpreted in light of differences in study design and methodological resolution. Population genomic analyses in medicinal plants vary widely in resolution: whole-genome resequencing studies (e.g., *F. suspensa* [17], typically *n* = 20–100 individuals) provide dense genome-wide SNP data suitable for selection scans, whereas reduced-representation approaches, such as RAD-seq (typical per-sample coverage 5–15×), trade genomic breadth for cost efficiency but may miss causal variants in unsequenced regions. Transcriptomic studies of environmental response commonly use *n* = 3–4 biological replicates per condition, which is adequate for detecting large-effect differentially expressed genes but may be insufficient for detecting subtle expression changes or formal genotype-by-environment interactions. Statistical thresholds are inconsistent across studies: significance cutoffs for differential expression range from FDR < 0.05 to nominal *p* < 0.01, and GWAS significance thresholds vary depending on marker density and population structure correction. These differences make it difficult to compare results across studies and highlight the need to report sequencing strategy, sample size, replication, filtering criteria, and statistical thresholds, as discussed in Section 5.5.

The origins of botanical material are complicated, and adulteration often happens. On top of that, quality control is no easy task. Conventional tools that rely on morphology and a handful of chemical markers often fail to provide, especially when dealing with processed or powdered products. For species identification, DNA barcoding relies on conserved loci such as *matK* and *rbcL*. When it comes to telling closely related taxa apart, the ITS2 region works better [20,21]. Determining geographical origin involves more than just identifying which species one has. Markers like SNP and InDel stay stable over time and reflect the genetic differentiation found across different regions. When one brings in population genomic data, that signal gets even stronger, which gives researchers a reliable tool for tracing geographic origin [22]. Quality evaluation used to rely mostly on looking at physical features, but it has moved toward high-resolution genomic approaches. That said, people rarely put together data on species identification, geographic origin, and phytochemical quality in a single analysis. Integrative approaches that combine multi-omics data with machine learning can connect these different data layers and help make quality evaluation more consistent overall.

Translating these genomic insights into practical quality control systems requires a tiered strategy. First, species authentication can be achieved through the barcode markers discussed in Section 2.2. Second population-level origin assignment can be performed using a panel of geographically informative SNPs or InDels, with assignment confidence quantified by population genetic models. Third, chemotype prediction can link specific genetic variants (e.g., SNP haplotypes in biosynthetic gene clusters) to expected metabolite profiles through genome-wide association or genomic prediction models. When implemented as a sequential screening pipeline, such a system would enable quality evaluation that integrates genetic identity, geographic provenance, and predicted chemotype—an improvement over single-marker or single-metabolite approaches currently in use.

### 2.2. From Field to Sequence: Molecular Authentication and Origin Traceability

Taxonomic ambiguity and frequent adulteration are both common, and they undermine consistency in clinical outcomes. Sequence-based molecular techniques offer a viable alternative. These methods outperform morphology-based approaches in objectivity and reproducibility. The plastid loci *matK* and *rbcL* are widely used plant DNA barcodes, but in most relevant studies, the ITS2 region is the preferred marker. This region stands out because it has higher interspecific genetic variation. Moreover, it delivers stable, reliable amplification across diverse plant lineages [20,21]. Quality-controlled reference libraries and sequence databases are what make molecular methods work. With those resources, researchers can routinely compare the data to verified references—and that directly improves the reliability of species identification.

Currently, next-generation sequencing (NGS) has substantially broadened its role in molecular authentication. This is not just about identifying a single species anymore. DNA metabarcoding addresses this need for barcode amplification with high-throughput sequencing. What that means in practice is that multiple taxa can be detected at once, even in complex samples. This metabarcoding approach is effective for processed herbal products. It is also perfectly suitable for multi-component formulations. Compared with conventional morphological and chemical analytical methods, metabarcoding can still provide large-scale, reliable identification of species composition, and it is effective at detecting substitutions, contaminants, and any unlabeled ingredients. Commonly used analytical methods fail to detect these targets [23,24]. Part of the problem is that technical factors can introduce interference during detection. Primer bias, uneven amplification efficiency, and incomplete reference databases stand out as key issues. Therefore, rigorous experimental design and strict validation are a must.

Provenance authentication goes beyond species identification. It digs into the genetic structure of target populations. SNP and InDel markers capture genome-wide genetic differentiation, and that differentiation exists across geographically distinct populations. Chemical fingerprints are easily affected by environmental variation and post-harvest processing. DNA markers, by contrast, have stable genetic properties, which makes them considerably more reliable for origin traceability. SNP and InDel datasets, combined with population genetic models, allow us to resolve fine-scale population genetic structure. They also help infer gene flow dynamics and reveal region-specific allelic patterns, which provide a quantitative basis for origin assignment [22]. Recent studies have been making heavy use of reduced-representation sequencing (e.g., RAD-seq) and whole-genome resequencing. Reference panels for key medicinal species are now far more comprehensive, so geographic origin assignment achieves higher detection accuracy.

Integrating taxonomic authentication with chemical quality evaluation is a core objective—and a key focus in medicinal resource management. Genomic markers and metabolomic data are the tools that link genotype to chemotype. These associations help clarify population-level genetic variation, which underpins differences in bioactive compound composition. Organelle genomes, particularly chloroplast and mitochondrial DNA, provide complementary tools for lineage tracing and domestication analysis because of their conserved structure and maternal inheritance. Meanwhile, portable sequencing technologies are shifting authentication from the laboratory to the field, enabling near real-time, on-site verification throughout the supply chain.

Despite these advances, several limitations warrant consideration (Table 1). ITS2, while widely adopted for its high interspecific resolution, is subject to primer bias and amplification failure in degraded or highly processed herbal products [23,24]. Incomplete reference databases remain a persistent bottleneck: many medicinal species lack authenticated reference sequences, leading to ambiguous or incorrect assignments. For heavily processed formulations, DNA metabarcoding may fail to recover amplifiable DNA; in such cases, shotgun metagenomics or complementary chemical authentication methods may provide complementary alternatives.

Despite their utility, the practical application of organelle genomes (cpDNA and mtDNA) remains constrained by the lack of an integrated analytical framework, limiting regulatory standardization and routine quality control. Addressing this challenge requires harmonized genomic reference databases and standardized analytical workflows. Integrating multi-omics datasets through data-driven approaches will ultimately enable more consistent evaluation systems and support robust, reproducible quality assessment of medicinal materials.

### 2.3. Epigenetics and Metabolic Feedback: Closing the Regulatory Loop

Geo-authentic materials tend to have better phytochemical profiles. These profiles indicate that the regulatory networks behind secondary metabolism have shifted. To figure that out, researchers combine transcriptomic and metabolomic data and use them to build co-expression networks. What these networks do is link gene expression directly to metabolic traits, and by doing so, they help uncover candidate regulators that lie outside the well-trodden, established pathways [25]. As for the genes that code for rate-limiting enzymes involved in making terpenoids, flavonoids, and alkaloids, within those network modules, their expression turns out to be tissue-specific. Big changes in expression are not necessary to see major shifts in metabolic flux—small ones will suffice [26]. That said, transcriptional variation on its own still cannot explain why those stable, site-specific chemotypes keep showing up across different populations. Genetic variation plays a critical role. Methods like QTL mapping and GWAS help link observable traits—phenotypic traits—back to specific genomic regions. Tossing in multi-omics data [27] still does not fully resolve the problem. These approaches keep missing those dynamic, environment-dependent responses, and those responses are exactly what define geo-authenticity.

So, how does environmental information enter genetic systems? A big part of it works through epigenetic regulation. DNA methylation is a good example. It picks up on what is going on in the environment, and then it tweaks gene expression accordingly—but the key is that the underlying DNA sequence itself never changes [28]. Epigenetic changes do not operate on their own—they work through regulatory networks that are already in place. Those networks are the ones controlling how carbon and energy get allocated. The interesting part is that this regulation runs both ways. Why? Because metabolic intermediates can end up serving as substrates or cofactors for chromatin-modifying enzymes. This whole setup creates a feedback loop: metabolic activity keeps talking back to the epigenetic landscape, and vice versa [29]. So, when one sees those stable geo-authentic phenotypes, they represent the combined result of genetic variation, environmental conditions, and the metabolic state—not some fixed genetic program that runs on autopilot [30,31].

What we call geo-authentic quality does not arise from any single factor. It emerges from the interplay between genetic variation, epigenetic regulation, and metabolic feedback. Right now, generating data is not the bottleneck anymore. The principal challenge lies in model construction, because any decent model has to pull all these layers together, and on top of that, capture how they interact across different biological scales.

Geo-authentic quality depends on three interacting layers. Population-level genetic differentiation defines the possible range of chemotypes. Epigenetic modifications adjust transcriptional output to match local environmental conditions, and metabolic feedback loops then stabilize the phytochemical profile. Each layer comes with its own toolkit. Population genomics handles provenance tracing. Transcriptome-wide association studies pick out candidate regulators, and chromatin profiling captures epigenetic marks. So far, people have only started linking these tools together. That shifts the focus to biosynthetic pathways. These are the routes that take genetic and environmental instructions and turn them into the bioactive compounds—the very ones responsible for TCM’s therapeutic effects.

Experimental dissection of these regulatory loops requires a combination of complementary approaches. Bisulfite sequencing or whole-genome bisulfite sequencing (WGBS) can be used to map DNA methylation changes across environmental gradients and correlate them with metabolite profiles. ATAC-seq and ChIP-seq, for example, for H3K4me3, H3K27ac, and H3K27me3, can identify chromatin accessibility changes and active regulatory regions associated with biosynthetic gene expression. Perturbation of key metabolic enzymes (via CRISPR knockout or chemical inhibition) followed by measurement of chromatin marks can test whether specific metabolites act as cofactors for chromatin-modifying enzymes, thereby testing the proposed feedback loop. Integrating these assays with time-series transcriptomic and metabolomic data could provide the multi-scale evidence needed to establish causal epigenetic–metabolic connections in medicinal plants.

## 3. From Gene to Metabolite: Biosynthetic Logic of TCM Bioactive Compounds

Specialized metabolites, including terpenoids, alkaloids, and flavonoids, drive the therapeutic effects of medicinal plants. By overcoming the limitations of traditional biochemical approaches, genomics now enables direct interrogation of complex biosynthetic pathways [32]. Integrating genomic, transcriptomic, and metabolomic data associates candidate genes with metabolite accumulation and facilitates the identification of novel enzymes and regulatory factors [33].

Within biosynthetic gene clusters (BGCs), co-localized pathway genes share coordinated regulation, facilitating the discovery of previously uncharacterized biosynthetic pathways [34]. However, computational predictions require experimental validation, typically achieved through heterologous expression and metabolic engineering in microbial hosts [35]. Integrating multi-omics with synthetic biology further elucidates metabolite function and enables scalable production of plant-derived therapeutics [36].

### 3.1. Genome-First Pathway Discovery: Strategies and Tools

High-quality plant genomes and their annotation enable the identification of key enzyme families that underlie chemical diversification [37]. Co-expression and association analyses of integrated transcriptomic and metabolomic data link candidate genes to metabolite accumulation [38].

Analysis of high-dimensional genomic datasets increasingly relies on integrating machine learning with phylogenetic approaches, enabling the identification of specialized enzymes overlooked by conventional methods. Characterization of biosynthetic gene clusters (BGCs) further strengthens these predictions [34]. By restricting candidate genes to defined genomic intervals, BGC analysis narrows the search space and facilitates reconstruction of complete biosynthetic routes.

Experimental validation is essential for confirming predictions from computational and multi-omics analyses and typically relies on heterologous expression in microbial hosts, particularly yeast, to verify enzyme function and reconstruct biosynthetic pathways [39]. This integration of prediction and verification provides a practical framework for pathway characterization and supports the scalable production of plant-derived therapeutics.

Several bioinformatics tools support genome-first pathway discovery.The computational infrastructure for genome-first pathway discovery has matured considerably. For biosynthetic gene cluster (BGC) prediction, antiSMASH (antibiotics and Secondary Metabolite Analysis Shell) remains one of the most widely used tools, identifying BGCs from genomic sequences based on conserved domain architecture and gene neighborhood analysis. For co-expression network inference, WGCNA (Weighted Gene Co-expression Network Analysis) correlates transcriptomic modules with metabolite accumulation to nominate candidate biosynthetic genes. Machine learning methods have further expanded the toolbox: deep learning models can assist enzyme function prediction from protein sequence features, and graph neural networks can prioritize candidate genes within BGCs by integrating genomic context, co-expression, and evolutionary conservation signals. Recommended parameter settings and thresholds have been reviewed elsewhere, but key best practices include (i) using FPKM or TPM normalization for RNA-seq input, (ii) applying soft-thresholding power selection based on scale-free topology fit in WGCNA, and (iii) validating BGC predictions with comparative genomics across related species before undertaking heterologous expression.

### 3.2. Three Case Studies in Pathway Elucidation

The following three case studies—artemisinin, paclitaxel, and flavonoids—represent the best-characterized biosynthetic pathways in medicinal plants. Table 2 provides a comparative summary of their key features.

#### 3.2.1. Artemisinin—A Linear Pathway Resolved

**Figure 3:**
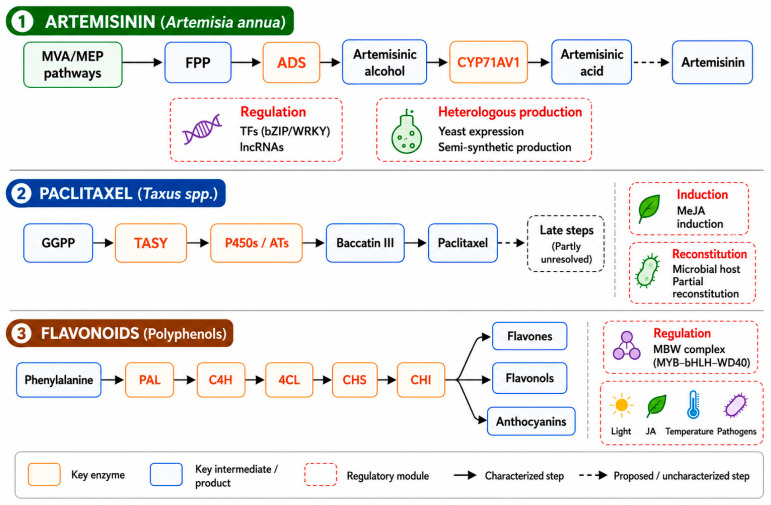
Biosynthetic organization of representative plant specialized metabolites. Comparison of biosynthetic routes for three major TCM bioactive compound classes. (**Top**) Artemisinin (sesquiterpene lactone) in *A. annua*. (**Middle**) Paclitaxel (diterpenoid) in *Taxus* spp. (**Bottom**) Flavonoid (polyphenols) branching from the phenylpropanoid pathway. Solid arrows indicate enzymatically characterized steps; dashed arrows indicate proposed or uncharacterized steps. Abbreviations: MVA, mevalonate; MEP, methylerythritol phosphate; FPP, farnesyl diphosphate; ADS, amorpha-4,11-diene synthase; CYP71AV1, cytochrome P450 monooxygenase 71AV1; TFs, transcription factors; bZIP, basic leucine zipper; WRKY, WRKY transcription factor family; lncRNAs, long non-coding RNAs; GGPP, geranylgeranyl diphosphate; TASY, taxadiene synthase; P450, cytochrome P450; ATs, acyltransferases; MeJA, methyl jasmonate; PAL, phenylalanine ammonia-lyase; C4H, cinnamate 4-hydroxylase; 4CL, 4-coumarate-CoA ligase; CHS, chalcone synthase; CHI, chalcone isomerase; MBW, MYB–bHLH–WD40; JA, jasmonic acid.

Artemisinin, a sesquiterpene lactone from *A. annua*, is a frontline antimalarial and a well-characterized example of plant specialized metabolism. The pathway begins with isoprenoid precursors supplied by the MVA and MEP pathways, which converge on FPP. ADS cyclizes FPP, and cytochrome P450 enzymes—principally CYP71AV1—oxidize the product to artemisinic acid [40]. A non-enzymatic photo-oxidative step then converts dihydroartemisinic acid into artemisinin. The entire route operates in glandular trichomes, whose spatial organization favors efficient metabolite accumulation. Terpenoid-related gene family expansion underpins this metabolic capacity (Figure 3) [30,43].

Control of this site-specific metabolic network operates at multiple levels. Transcription factors, particularly bZIP and WRKY family members, drive biosynthetic gene expression and shape secondary metabolite accumulation [47]. At the post-translational level, kinase-mediated phosphorylation modulates transcription factor activity, enabling rapid responses to developmental and environmental cues [28]. Long noncoding RNAs (lncRNAs) contribute to transcriptional regulation and are linked to metabolic trait variation among *A. annua* genotypes [53].

Despite the landmark achievement of semi-synthetic artemisinin production in engineered yeast [40,52], the economic viability of this route has remained context-dependent. The market price of artemisinin has fluctuated substantially over the past two decades, driven primarily by supply-and-demand dynamics of plant-derived material [52]. Semi-synthetic production, which achieved titers of 25 g/L artemisinic acid [40], must compete economically with plant-derived supply, and its commercial viability depends on the relative cost of the two production routes at any given time. These market dynamics, combined with the capital expenditure required for stainless-steel fermentation infrastructure, underscore that technical feasibility does not guarantee economic viability. Regulatory considerations, including the requirement that semi-synthetic artemisinin meet identical purity specifications as the plant-derived product and navigate the FDA’s Drug Master File system, add further complexity to the commercialization pathway.

#### 3.2.2. Paclitaxel—A Branched Pathway with Missing Steps

Paclitaxel, a structurally elaborate diterpenoid from *Taxus* species, is an antitumor compound and one of the most complex plant secondary metabolites known. Taxadiene synthase initiates biosynthesis by cyclizing GGPP to taxa-4(5),11(12)-diene—the first committed step in taxane biosynthesis [41]. Roughly twenty enzymatic transformations follow [54]. Cytochrome P450 monooxygenases and acyltransferases account for most of these modifications, including hydroxylation and acylation reactions leading to intermediates such as baccatin III [44,55]. Although taxadiene synthase and several hydroxylases have been characterized [56], the paclitaxel biosynthetic pathway has historically been difficult to resolve, with several mid- and late-stage oxidation, acylation, and oxetane ring-forming steps remaining incompletely characterized. Recent enzyme characterization studies have clarified several previously unresolved steps in the paclitaxel pathway. Martinelli et al. [46] reported the functional characterization of final cytochrome P450-mediated oxidation steps, and co-expression analysis combined with virus-induced gene silencing in Taxus suspension cells has helped identify candidate acyltransferases involved in C2 and C10 side-chain modifications. The formation of the oxetane ring, the defining structural feature of paclitaxel, has also been proposed to proceed through an epoxide intermediate catalyzed by a CYP450 enzyme, although direct biochemical confirmation is still required. Paclitaxel biosynthesis is more branched than the linear artemisinin route, and this branching has made complete heterologous reconstruction a persistent challenge (Figure 3).

The control of paclitaxel biosynthesis depends not only on the enzyme-catalyzed steps but also on where the compounds are located within Taxus tissues and what developmental stage those tissues have reached. The metabolic flux in this pathway goes hand in hand with tissue differentiation and can shift in response to external signals, especially methyl jasmonate. In Taxus cell cultures, methyl jasmonate is a standard go-to elicitor for triggering taxane production [44]. Currently, multi-omics (genomics, transcriptomics, and metabolomics) provide higher-resolution insights at pathway details [46]. With these datasets, we can nail down which enzyme steps are missing. They also show that the regulation is spread across many different genes, not just one single master switch.

Synthetic biology gets around the physiological and regulatory limits that come with natural Taxus systems. By rebuilding the early steps of the paclitaxel pathway in microbial hosts, researchers can test out enzymes and crank out taxane precursors [54]. Achieving complete heterologous biosynthesis will require characterizing the remaining enzyme steps and determining how the regulatory mechanisms control the pathway’s activity. Once the entire pathway is reconstructed, that sets the stage for a sustainable supply of this clinically important compound. Complete heterologous reconstitution of the paclitaxel pathway in a microbial host has not yet been reported, partly because several late-stage enzymes require an endoplasmic reticulum membrane environment and specific redox partner proteins that are difficult to reproduce in prokaryotic chassis. Structural studies of pathway enzymes or multi-enzyme complexes, including cryo-EM-based approaches, may help assign the remaining enzymatic steps and support more rational pathway engineering.

#### 3.2.3. Flavonoids—A Network of Branching Decisions

Flavonoids make up a big family of polyphenolic compounds, and they come with all sorts of biological activities. These compounds all trace back to the phenylpropanoid pathway. In that pathway, three key enzymes—phenylalanine ammonia-lyase (PAL), cinnamate 4-hydroxylase (C4H), and 4-coumarate: CoA ligase (4CL)—work together to turn phenylalanine into p-coumaroyl-CoA, which is the main precursor for making flavonoids [45]. Next, chalcone synthase (CHS) and chalcone isomerase (CHI) handle the condensation and cyclization steps, producing naringenin. This intermediate serves as the branch point for the main flavonoid subclasses—flavones, flavonols, and anthocyanins. The genes involved in biosynthesis split into two functional groups: early ones for building the core scaffold, and late ones for further structural tweaks [57]. Upon knockout of key enzymes like CHS or CHI, flavonoid production grinds to a halt, and metabolic flux gets rerouted into related phenylpropanoid pathways, demonstrating the tight connectivity of this system [51].

A set of conserved transcription factor complexes—especially the MBW complex (made up of R2R3-MYB, bHLH, and WD40 proteins)—drives much of the flavonoid metabolic flux by turning on anthocyanin biosynthesis genes. This regulatory setup helps explain why metabolic shifts depend on context, like how wounding triggers anthocyanin buildup in poplar [58], or how carbon gets rerouted toward catechin in tea plants [57]. Multi-omics data have expanded our view of this picture. Take chrysanthemum as an example—combined analyses show that both shifts in gene expression and gene diversification at the family level help shape the structural variety of flavonoids seen across different medicinal species (Figure 3) [45].

Flavonoid metabolites are so complex, and this complexity arises from two principal sources. One is the sheer variety of enzymes involved, and the other is how tightly the gene expression networks are coordinated. Once one has a handle on these regulatory systems, it directly provides clues for metabolic engineering. It also helps breed medicinal plants that have better, more tailored flavonoid profiles. The core flavonoid pathway is well resolved; however, species-specific tailoring steps—including tissue-specific glycosylation, methylation, and acylation patterns that determine bioactivity—are far less understood and represent a priority for future functional characterization.

### 3.3. Transcriptional Control to Chromatin: The Regulatory Hierarchy

Several regulatory layers—including transcription factors, signaling pathways, and environmental cues—control how specialized metabolites build up in plants. Transcription factor complexes like the MBW complex (MYB, bHLH, and WD40 proteins) turn on flavonoid biosynthesis genes and help steer metabolic intermediates down different branch pathways [42,48]. Similar regulatory players show up elsewhere. ORCA3 in *Catharanthus roseus* drives alkaloid biosynthesis, and in *A. annua*, AabZIP1 promotes artemisinin production [12,59].

Phytohormone signaling operates above the transcriptional layer, linking developmental and environmental inputs to metabolic responses. In jasmonate (JA) signaling, degradation of JAZ repressors releases MYC2, which activates downstream metabolic genes, including those for nicotine biosynthesis in tobacco [60,61]. JA signaling converges with ethylene and salicylic acid pathways to form an interconnected network, not a single linear route [62].

Chromatin-level regulation adds another dimension to transcriptional control. DNA methylation and histone modifications respond to developmental and environmental signals, altering the accessibility of biosynthetic gene loci to the transcriptional machinery [63,64]. Assays for transposase-accessible chromatin with sequencing (ATAC-seq) map genome-wide chromatin accessibility, identifying regulatory regions activated or silenced across conditions. Chromatin immunoprecipitation followed by sequencing (ChIP-seq) for histone modifications—such as H3K4me3 (active promoters), H3K27ac (active enhancers), and H3K27me3 (repressed regions)—can help define the epigenetic states of biosynthetic genes. Practical challenges include the requirement for fresh tissue, the need for high-quality antibodies validated in the target species, and the computational difficulty of mapping reads to complex, often polyploid, medicinal plant genomes. Integrating ATAC-seq and ChIP-seq with transcriptomic and metabolomic time-series data has not yet been widely applied for identifying the chromatin-level control points of bioactive compound biosynthesis.

Two decades of work on artemisinin, paclitaxel, and flavonoids point to a shared regulatory architecture for specialized metabolism. Transcription factor complexes set the baseline. Hormone signals adjust it. Chromatin structure gates access to the genes. As a result, the critical bottleneck has moved from gene discovery to functional proof. Knowing that a cytochrome P450 gene clusters together with a terpene synthase is not equivalent to knowing that it performs the expected oxidation. What comes next shifts us from analysis to building things. Section 4 deals with how to take these biosynthetic blueprints and put them back together in heterologous hosts, thereby linking computational prediction directly to experimental verification.

## 4. Engineering Production: Synthetic Biology for TCM Natural Products

Plant genomics and synthetic biology have pushed the study of medicinal plant resources beyond descriptive characterization. Current efforts aim to link genetic information with metabolite production in a clear, stepwise fashion.

A central challenge of determining how to integrate diverse omics data types remains. Genomic, transcriptomic, and metabolomic datasets differ in scale and resolution, and metabolite levels rarely match gene expression directly. Machine learning now steps in to handle this complexity by analyzing multi-layered datasets. It can pick out regulatory genes that would slip past single-data-type analyses. Turning computational pathway predictions into functional in vivo systems remains a big hurdle for synthetic biology. In silico models lay out complete pathways, but plenty of steps still lack experimental characterization. Reconstructed pathways in heterologous hosts encounter practical obstacles, including metabolic imbalances and incompatibilities between plant enzymes and microbial hosts.

These challenges require iterative optimization under the Design–Build–Test–Learn (DBTL) framework. Spatial omics and chassis engineering now provide sharper tools for pathway reconstruction.

Systems-level integration of phytochemistry and metabolic engineering now allows for more controlled, scalable production of botanical therapeutics. This approach reduces reliance on wild harvesting and avoids the inconsistency inherent to conventional agriculture, securing a steadier supply of important compounds such as artemisinin and paclitaxel [65,66]. Metabolic engineering also broadens our access to plant-derived molecules.

### 4.1. Building the Chassis: Heterologous Expression Systems

**Figure 4:**
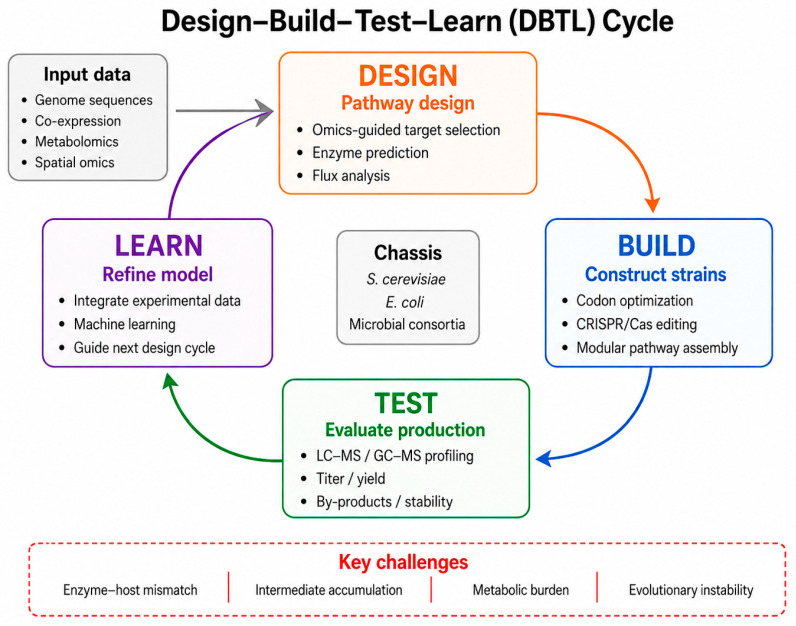
The DBTL cycle (Design–Build–Test–Learn) applied to microbial production of plant natural products. Design: genome sequences, co-expression networks, and metabolomic profiles drive pathway selection, enzyme prediction, and biosynthetic gene cluster mining. Build: candidate pathways are assembled in microbial chassis using codon optimization, CRISPR/Cas editing, and modular regulatory elements. Test: engineered strains undergo metabolomics screening (LC–MS or GC–MS). Learn: experimental data integrated with machine learning and flux analysis refine enzyme selection and process conditions. Challenges include enzyme–host mismatch, intermediate accumulation, metabolic burden, and laboratory-to-industrial scale-up. Colors distinguish the major stages of the DBTL cycle: orange, Design; blue, Build; green, Test; and purple, Learn. The gray boxes indicate input data and chassis information, and the red dashed box summarizes key challenges associated with the DBTL workflow. Abbreviations: LC–MS, liquid chromatography–mass spectrometry; GC–MS, gas chromatography–mass spectrometry; CRISPR/Cas, clustered regularly interspaced short palindromic repeats/CRISPR-associated protein system.

Porting plant biosynthetic pathways into microbial systems is now a central strategy for producing plant-derived natural products. *Saccharomyces cerevisiae* and *E. coli* are the predominant hosts for this work, because their genomes are well characterized and genetic engineering tools are readily available (Figure 4) [67,68]. Host selection follows the biochemical demands of the target pathway. Yeast excels at expressing plant cytochrome P450 enzymes because its internal membrane systems support proper enzyme localization and electron transfer [65]. *E. coli*, by contrast, serves well for rapid testing of individual enzymatic steps and for boosting precursor supply [49]. A decision framework for matching pathway features to the appropriate microbial chassis is provided in Table 3.

Beyond the choice of primary chassis, several fundamental biological limitations constrain microbial production of plant natural products. First, many plant cytochrome P450 enzymes—which catalyze key oxygenation steps in terpenoid, alkaloid, and flavonoid biosynthesis—require specific cytochrome P450 reductase (CPR) partners and integrate into the endoplasmic reticulum membrane. Prokaryotic hosts (*E. coli*) lack ER, and while P450s can be expressed with engineered N-terminal modifications, catalytic efficiency is often reduced by 10- to 100-fold compared with the native plant context. Second, plant-specific post-translational modifications—including complex N-glycosylation patterns, proline hydroxylation, and disulfide bond formation—are absent or incompletely recapitulated in microbial systems, which can affect enzyme folding, stability, and activity. Third, the supply of non-proteinogenic precursor metabolites (e.g., non-canonical amino acids for alkaloid biosynthesis, benzoyl-CoA for taxol side chains) may require engineering of additional heterologous pathways, compounding metabolic burden. Fourth, product toxicity can further limit production because many specialized metabolites evolved as chemical defense compounds and may inhibit microbial growth at high intracellular concentrations. Addressing these limitations through protein engineering (directed evolution of P450-CPR pairs), organelle engineering (peroxisomal or vacuolar compartmentalization in yeast), and dynamic pathway regulation [69,70] will be important for improving microbial production of TCM-related natural products.

Yeast-based artemisinin precursor biosynthesis required heterologous expression of plant enzymes (ADS and CYP71AV1) and re-engineering of the mevalonate pathway to ensure sufficient FPP supply [65]. As microbes lack the final photochemical conversion step, they are coupled with a chemical finishing process, forming a hybrid strategy that enables industrial-scale manufacturing [40].

Long, energy-intensive heterologous pathways often overburden single-host systems, restricting growth and product yields. To overcome this, synthetic microbial consortia distribute pathway modules across distinct strains [71], enhance pathway stability, and mitigate enzyme–host incompatibilities, as well as intracellular interference. Physiological incompatibilities between eukaryotic plants and prokaryotic hosts often limit heterologous pathway expression. In microbial systems, deviations from native protein folding environments, cofactor availability, and intracellular organization frequently render plant enzymes unstable or inactive. Cytochrome P450s rely on membrane-associated electron transfer systems that are not natively present in microbial hosts [40,65]. Addressing these limitations requires pathway optimization strategies such as codon optimization, protein fusion, and host metabolic engineering [49].

**Table 3:** Decision guide for microbial chassis selection.

Pathway Feature	Recommended Chassis	Rationale
Plant terpenoids (C15, C20) [40,65,66]	*S. cerevisiae*	Native MVA pathway; ER for P450 expression
Bacterial polyketides [67]	*Streptomyces* spp.	Native precursor pools; established genetic tools
Simple plant phenolics [49]	*E. coli*	Rapid growth; well-characterized metabolism
Complex alkaloids [50,71]	*S. cerevisiae* or co-culture	Compartmentalization; pH control
Membrane-bound P450 enzymes [65]	*S. cerevisiae*	Endomembrane system; closer to plant context
Industrial-scale [67,68]	*E. coli* or *Corynebacterium glutamicum*	High-density fermentation; GRAS status

### 4.2. The DBTL Cycle: Iterative Pathway Optimization

In the Design phase, in silico pathway models should be calibrated against experimental flux measurements. The Build phase benefits from automated platforms capable of constructing large variant libraries in parallel. During the Test stage, target metrics depend on the compound class: for high-value pharmaceuticals, titers in the mg/L–g/L range represent meaningful early milestones, while for commodity chemicals, higher titers and yields are required for economic viability. The Learn phase should quantify improvement per cycle; leading implementations have demonstrated multi-fold titer increases through iterative DBTL optimization [72]. These benchmarks, while context-dependent, provide a practical framework for evaluating progress and comparing platforms across laboratories.

Metabolic engineering extends beyond inserting heterologous genes into a host. The host cells also have to rethink how they split carbon and energy between their own native metabolism and the engineered one. How they shift those resources around determines whether the pathway will produce sufficient quantities. A common strategy involves boosting precursor pools and dialing down competing pathways that run inside the cell. In *E. coli* carrying the mevalonate (MVA) pathway, a higher supply of FPP improved terpenoid production by lifting substrate limitation [73]. Researchers strengthen upstream pathways and attenuate competing branches to direct more flux toward the target product [49]. However, excessive flux rerouting may compromise host growth and genetic stability. Modular pathway design can address this limitation by separating the engineered route into a precursor-supply module and a downstream-conversion module. This architecture enables independent optimization of each module, thereby reducing metabolic burden and improving overall production efficiency [49,74].

Metabolic engineering has come a long way from just cranking up the expression of one enzyme at a time. Currently, metabolic engineering fine-tunes the expression of multiple steps along a pathway to keep the whole metabolism in balance, providing substantially tighter control over product formation than with single-gene strategies. Synthetic regulatory elements, together with improved DNA assembly methods, let researchers adjust several genes within a pathway in a coordinated manner [75]. Inducible and population-dependent systems, such as quorum sensing, activate gene expression only under defined cellular conditions. This tactic resolves the common conflict between cell growth and secondary metabolite production that limits many microbial biosynthesis processes [69,76].

CRISPR/Cas genome editing has sharply accelerated the redesign of microbial production systems. Unlike earlier single-gene interventions, Cas nucleases hit multiple genetic loci simultaneously, giving better coordinated control of metabolic pathways and cellular flux distribution [70]. Metabolic engineering has thereby moved beyond stepwise gene insertion to integrated, genome-level pathway optimization [77]. Modern phytochemical biosynthesis now rests on the combined use of pathway-level regulation, inducible expression systems, and genome-scale engineering, which together raise the efficiency and reliability of microbial production of complex natural products.

### 4.3. From Lab to Bioreactor: Scaling Challenges and Economic Reality

Currently, engineered microbial systems operate well beyond proof-of-concept. Multi-step plant pathways are being reconstituted in yeast and bacteria, and producing terpenoids, polyphenols, and selected alkaloids at meaningful titers. Artemisinic acid production in S. cerevisiae set the benchmark: iterative strain and fermentation optimization raised titers from trace amounts to commercially relevant levels [40,65]. Similar strategies have since been extended to resveratrol, various isoprenoids, and a growing list of structural analogs not accessible from native plants [50,78]. However, translating laboratory successes to industrial deployment faces three persistent barriers. The first is enzyme–host mismatches. Cytochrome P450s are central to plant specialized metabolism, but they rely on membrane environments and electron transfer partners that microbial hosts simply lack [65]. Directed evolution and protein fusion constructs have narrowed this gap for some enzymes, but a general solution applicable across diverse enzyme classes remains elusive. The second barrier is metabolic burden. High-flux heterologous pathways compete with native metabolism for carbon, energy, and redox cofactors, resulting in a trade-off between product formation and cell growth [49]. Dividing pathways across synthetic microbial consortia relieves some of this load by distributing enzymatic steps among specialized strains [79]. Meanwhile, inducible and quorum-sensing regulatory systems allow one to temporally separate biomass accumulation from metabolite production [71,76]. The third barrier is evolutionary. During extended cultivation, selection ends up favoring variants that silence or delete the engineered pathway, which steadily erodes productivity [80].

Economic feasibility adds another constraint. Microbial biomanufacturing competes directly with extraction from cultivated plants. For industrial adoption, one must meet the minimum thresholds for titer, yield, and productivity, and many engineered strains still fall short of those [73,81]. Costs build up across substrate, reactor operation, and downstream purification, so strain improvements alone will not ensure economic viability. Progress demands co-optimizing the biological system and the fermentation process together, not sequentially.

A more fundamental barrier is pathway incompleteness. For alkaloids, complex terpenoids, and other high-value natural products, too many enzymatic steps and regulatory elements are still missing or uncharacterized. Full reconstruction, therefore, remains unfeasible in any heterologous host [82]. These gaps are not conceptual—the underlying chemistry is clear. They reflect a deficit in functional annotation. Fortunately, genome mining, co-expression analysis, and machine-learning-based enzyme function prediction are steadily closing that deficit [72,83]. The emerging workflow uses multi-omics data and computational modeling to prioritize targets ahead of the DBTL cycle—moving strain design from empirical trial-and-error to hypothesis-driven strategies. Directly linking computational prediction with experimental validation in microbial systems accelerates pathway optimization, and at the same time supplies the functional data needed to verify biosynthetic models from the previous chapter. With the production pipeline in place, the remaining question is the therapeutic effects of these compounds, which is the focus of Section 5.

Translating microbial biomanufacturing from the laboratory to industrial production requires attention to regulatory, biosafety, intellectual property, and economic constraints. For pharmaceutical applications, fermentation-derived products must meet regulatory requirements for purity, potency, consistency, and process-related impurities. For food or supplement applications, additional safety evaluation and market-specific authorization may be required. Biosafety considerations include containment of genetically modified production strains, prevention of unintended release, and avoidance of horizontal gene transfer, according to the host strain, introduced pathway, and local biosafety regulations. Intellectual property considerations may also affect commercialization, because engineered strains, pathway designs, enzyme variants, and fermentation processes can be protected separately. Economic feasibility further depends on downstream purification, which can account for a substantial fraction of total production cost, as well as compliance with Good Manufacturing Practice (GMP) requirements for pharmaceutical-grade products.

## 5. Systems Pharmacology of Traditional Chinese Medicine

Chinese herbal compound prescriptions exert their therapeutic effects by simultaneously acting on multiple biological targets through various chemical components. The target experimental model is simply unable to fully capture this mode of action. The combination of multi-omics approaches with network pharmacology provides the corresponding tools to investigate how these compounds affect interrelated signaling pathways, immune responses, and metabolic processes [1,58,84]. Therefore, the field must move from descriptive network maps toward a causal understanding of the mechanism of action. Beyond intracellular signaling networks, intercellular communication—mediated by extracellular vesicles (EVs) such as exosomes—provides an additional, non-genomic mechanism by which multi-component interventions can propagate system-level effects. Epithelial cell-derived exosomes carry miRNA and protein cargos that are taken up by recipient cells and alter their transcriptional and functional state [85], offering a concrete example of how a single cellular source can simultaneously modulate multiple targets in distal cell populations—a mode of action conceptually analogous to the distributed effects of TCM formulations. A fundamental principle of systems biology is that disease processes should be analyzed through reconstructed interaction networks [86].

### 5.1. Network Pharmacology: From Single Target to System-Level Logic

Network pharmacology redefines the action of drugs as a distributed process: rather than a single compound acting on a single target, multiple components jointly participate in numerous nodes across multiple interconnected biological networks [1]. The therapeutic effect stems from the collective effect of these distributed disturbances, but when combined, they are sufficient to reshape the pathway activity. Multi-component intervention measures can improve the systemic response and limit the development of drug resistance [87].

Analyzing complex TCM compound formulas involves integrating a compound library, target prediction, and protein interaction networks [88,89]. This approach shifts the focus of analysis from individual targets to functional protein modules. The herbal components converge on the signaling module centered around NF-κB [90]. In the field of oncology, multi-component compound preparations can simultaneously act on the PI3K-Akt and MAPK pathways; however, there are differences in the magnitude and consistency of their effects among different experimental models and compound preparations. Predicted targets must be verified through experiments and functional data.

**Figure 5:**
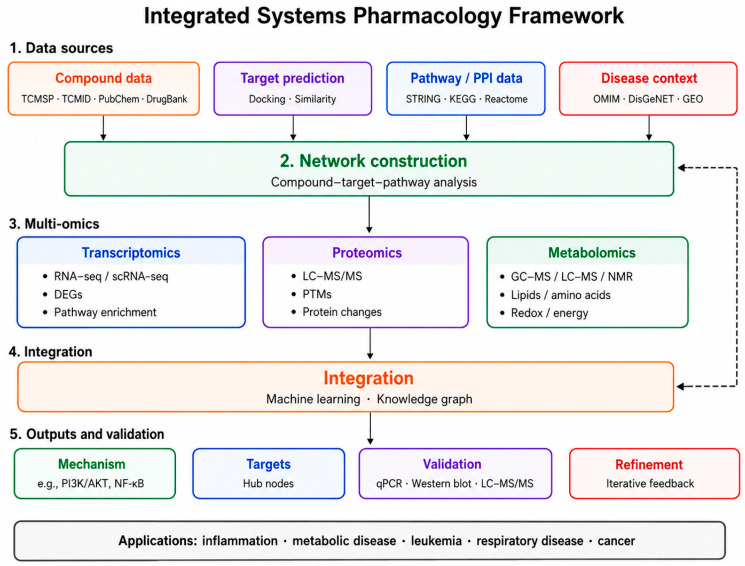
Integrated systems pharmacology framework for deciphering herbal mechanisms. Chemical profiles from public repositories (TCMSP, TCMID, PubChem, DrugBank) are screened against protein targets, and predicted interactions are contextualized within protein–protein interaction and pathway networks mapped onto disease-relevant gene sets. Multi-omics datasets—transcriptomic (RNA-seq, scRNA-seq), proteomic (LC–MS/MS), and metabolomic—are integrated to reconstruct multilayer regulatory architectures. Prioritized mechanisms undergo validation by qPCR, Western blotting, and LC–MS/MS profiling with an iterative feedback loop between computational prediction and empirical evidence. Different colors are used to distinguish the major data sources, omics layers, integration steps, and output/validation modules in the framework. Abbreviations: TCMSP, Traditional Chinese Medicine Systems Pharmacology Database and Analysis Platform; TCMID, Traditional Chinese Medicine Integrated Database; PPI, protein–protein interaction; OMIM, Online Mendelian Inheritance in Man; GEO, Gene Expression Omnibus; RNA-seq, RNA sequencing; scRNA-seq, single-cell RNA sequencing; DEGs, differentially expressed genes; PTMs, post-translational modifications; NMR, nuclear magnetic resonance; qPCR, quantitative polymerase chain reaction; PI3K, phosphoinositide 3-kinase; AKT, protein kinase B; NF-κB, nuclear factor kappa B.

Graph-based metrics, such as degree centrality and betweenness centrality, can be used to quantify the ability of individual nodes to regulate information flow within disease interaction networks [91]. Combining transcriptomics maps with perturbation-based gene characteristics enables the above analysis to expand from static network descriptions to context-dependent evaluations [92]. They may serve as both the convergence points for disease progression and drug responses [93] (Figure 5).

Despite its conceptual appeal, network pharmacology as commonly practiced in TCM research has several limitations and reproducibility concerns that should be explicitly addressed. First, docking-based and chemical-similarity-based target prediction algorithms can have substantial false-positive rates. The accuracy of top-ranked predictions depends on the quality of protein structures, scoring functions, and benchmark datasets, and may be further reduced when homology-modeled rather than experimentally determined protein structures are used. Second, many published TCM network pharmacology studies rely exclusively on in silico predictions without orthogonal experimental validation, which limits the reliability of their mechanistic claims. Third, PPI network topology is sensitive to database version, species annotation, and confidence-score thresholds, meaning that the same compound–disease pair can yield markedly different hub targets depending on analytical parameters. To improve reproducibility, the field should adopt minimum reporting standards: (i) specify database versions and access dates; (ii) report confidence-score thresholds for PPI edges; (iii) use independently validated target sets (e.g., DrugBank, ChEMBL) as positive controls; and (iv) follow computational predictions with at least one orthogonal validation method, such as chemoproteomics (e.g., thermal proteome profiling, activity-based protein profiling) or biophysical assays (e.g., surface plasmon resonance, microscale thermophoresis). A recommended workflow—from prediction through prioritization to validation—is provided in Figure 5.

### 5.2. Multi-Omics Dissection of TCM Mechanisms

Integrated omics captures the biological response to TCM intervention. Transcriptomic data reveal early gene expression shifts, while proteomic and metabolomic data reflect the downstream biochemical consequences [94]. Analyzing these layers together improves mechanistic interpretation [95,96]. Projecting this heterogeneous data into a shared mathematical space is one approach [97]. However, omics datasets differ in measurement scale, noise structure, and feature space, so dedicated integration algorithms are required. Multi-Omics Factor Analysis (MOFA) projects multiple omics layers into a shared latent space, identifying factors that explain coordinated variation across data types. DIABLO (Data Integration Analysis for Biomarker discovery using Latent cOmponents), implemented in the mixOmics R package, extends partial least squares to multi-omics classification and identifies feature panels that discriminate between treatment groups. For network-level integration, iOmicsPASS scores subnetworks by aggregating multi-omics signals within predefined biological modules, while graph neural network-based methods (e.g., MODA) learn latent representations of molecular entities by integrating genomic, transcriptomic, proteomic, and metabolomic interaction networks [98]. Bayesian frameworks and similarity network fusion provide complementary approaches suited to smaller sample sizes. The choice of method depends on the study design: MOFA and DIABLO are better suited to studies with sufficient sample sizes and well-matched omics layers, whereas graph-based methods excel when prior knowledge of molecular interaction networks is available.

Although not a TCM study, a recent study of miR-10b-5p in the diabetic cornea provides a useful methodological example of such an integrated pipeline: RNA-seq identified differentially expressed transcripts, LC-MS/MS-based proteomics quantified corresponding protein-level changes, overlap analysis prioritized concordantly regulated candidates, and biochemical assays (Western blot, activity measurements) confirmed the predicted regulatory mechanism under oxidative stress conditions [99]. This study illustrates how sequentially linked transcriptomic, proteomic, and functional validation steps can move from descriptive profiling to mechanistic confirmation.

Several experimental design considerations are important for generating interpretable multi-omics data. Regardless of the specific omics technologies employed, several design principles are critical for generating interpretable multi-omics data. Treatment and control groups should be randomized, and the sample processing order should be randomized to avoid batch confounding. Adequate biological replication is essential for statistical power, whereas technical replicates alone are insufficient. Matched time points across omics layers are critical for temporal alignment: transcriptomic and proteomic samples should be collected from the same animals at the same post-treatment intervals, as mRNA–protein correlation varies with time lag. For LC-MS/MS studies, pooled QC samples should be injected regularly throughout the analytical run to monitor and correct for instrument drift. Data normalization should be method-appropriate: for RNA-seq, DESeq2 or edgeR with their built-in normalization; for label-free proteomics, median or quantile normalization; and for metabolomics, probabilistic quotient normalization or ComBat for multi-batch studies. The use of stable isotope-labeled internal standards in metabolomics and spiked-in reference peptides in proteomics further improves quantitative accuracy and cross-study comparability.

#### 5.2.1. Transcriptomic Signatures

RNA sequencing is now the default first-pass assay for figuring out transcriptional responses to pharmacological interventions. The choice of transcriptomic platform determines the granularity of mechanistic insight. Bulk RNA-seq, the most widely used approach, provides averaged gene expression profiles across tissue homogenates but masks cellular heterogeneity. Single-cell RNA-seq (scRNA-seq) resolves transcriptional programs at an individual-cell resolution, enabling identification of rare responding cell populations and cell-state transitions during TCM treatment [100,101]. Spatial transcriptomics further preserves tissue architecture, mapping gene expression to histological context—particularly valuable for studying TCM effects on tissue-resident immune cells and compartment-specific metabolic reprogramming. Each platform entails distinct trade-offs: bulk RNA-seq offers lower cost and higher throughput; scRNA-seq requires fresh tissue and specialized computational pipelines; and spatial transcriptomics currently has lower transcript coverage than bulk or single-cell RNA-seq in many platforms. Combining these approaches—for example, using scRNA-seq to resolve cell-type-specific responses and validating spatial patterns in tissue sections—provides the most comprehensive transcriptional picture of TCM pharmacology.

Then, differential expression analysis, combined with pathway enrichment, maps out the signaling networks and gene sets modulated by TCM treatments. Inflammatory reprogramming in macrophages shows how multi-layered these responses are: upon stimulation, enhancer remodeling and chromatin accessibility changes drive broad gene activation [102], while NF-κB-dependent transcriptional elongation controls the timing and magnitude of transcript production for a subset of target genes [103]. The regulatory logic, in other words, distributes control across initiation, elongation, and chromatin state—not a single checkpoint.

Macrophage transcriptional responses are shaped by signal-dependent transcription factor networks that encode context-specific immune programs—a regulatory logic that complex herbal interventions also engage [104]. Oxidative stress triggers a stereotyped antioxidant transcriptional response: HMOX1, NQO1, and SOD2 are consistently upregulated across experimental systems [105]. Single-cell and time-resolved RNA-seq studies have since added a critical temporal dimension to this picture. Early activation is dominated by inflammatory and stress-response programs; later phases shift toward metabolic recalibration and tissue repair [100,101], and because these transitions depend on the cellular state, a single gene-expression snapshot is insufficient to capture the whole course of a TCM intervention. This represents a key functional gap: how can dynamic transcriptional trajectories be connected to specific therapeutic outcomes in traditional medicine? To bridge that gap, it is essential to pair time-series transcriptomics with proteomic and metabolomic readouts and ensure all measurements are taken at matched time points.

#### 5.2.2. Proteomic and Metabolomic Readouts

Protein abundance, enzymatic activity, and post-translational modifications introduce regulatory layers that transcription alone cannot reveal. High-resolution mass spectrometry now makes it possible to systematically compare proteomic and transcriptomic trends, thereby identifying cases where post-transcriptional regulation dominates. Peroxiredoxins represent a clear example of this disconnect. Because they are highly sensitive to redox state, they serve as central reporters of oxidative conditions. However, their functional behavior, including the shift from peroxidase activity to chaperone-like oligomeric assemblies, depends on structural transitions that cannot be captured by structural transcriptomics [106]. Peroxiredoxin function depends on redox-sensitive structural transitions. As oxidation levels rise, specific peroxiredoxin isoforms reorganize—moving from low-molecular-weight, peroxidase-active forms into higher-order oligomeric assemblies that then gain chaperone-like activity. Jang et al. [107] demonstrated that this structural rearrangement stabilizes unfolded proteins during stress, directly linking redox state to proteostatic adaptation. This functional plasticity operates along a continuum rather than as a binary switch, with the precise activity state determined by local oxidation potential and cellular context. More broadly, protein-level regulation, including oligomeric state and post-translational modification, encodes functional information that transcript-level measurements do not. For TCM research, that means proteomic profiling is not merely confirmatory, but essential for capturing regulatory complexity that transcriptomics alone misses. Proteomic studies in cancer and chronic inflammation. They consistently show coordinated changes across mitochondrial respiratory chain subunits and antioxidant protein networks. It suggests that energy metabolism and redox regulation are closely coupled—they do not operate independently [108]. Metabolomic data take these findings down to the small-molecule level. For example, changes in TCA cycle intermediates, fatty acid profiles, and glutathione synthesis represent the biochemical fallout from upstream regulatory events [109,110]. In principle, the three data layers could be linked—transcriptional regulation, proteomic machinery, and metabolomic output—into a single causal chain. However, to turn that principle into a disease-relevant account, there needs to be a solid case. Leukemia provides this, and similar patterns appear elsewhere: combined transcriptomic and metabolomic profiling of radiation injury, for instance, reveals coordinated changes in amino acid and lipid metabolism together with inflammatory signaling—showing that metabolic and immune responses are interdependent, not independent [111].

Analytical variability in LC-MS/MS-based proteomics and metabolomics represents a significant and often underappreciated source of irreproducibility in TCM multi-omics studies. Batch effects arising from sequential sample preparation, column aging, and instrument drift can confound biological signals, particularly in large cohort studies. Best practices include randomized sample processing order, inclusion of pooled quality control (QC) samples injected at regular intervals, and post-acquisition normalization using algorithms such as ComBat, robust LOESS regression, or quantile normalization. In proteomics, data-independent acquisition (DIA) has largely supplanted data-dependent acquisition (DDA) for quantitative studies owing to its superior reproducibility, lower missing-value rates, and broader dynamic range in complex samples. For metabolomics, the use of stable isotope-labeled internal standards and reporting metabolite identification confidence levels (as defined by the Metabolomics Standards Initiative) is essential for cross-study comparability.

### 5.3. Case Study: Multi-Omics Dissection of TCM Pharmacological Mechanisms

Table 4 summarizes the current state of omics-based evidence in TCM–leukemia research and highlights the gaps that remain to be addressed. Although leukemia-specific multi-omics studies remain limited, case studies from respiratory disease research illustrate how pharmacological multi-omics can move beyond conceptual network descriptions. One recent example is Keke Tablet (KKP), a traditional herbal preparation used for respiratory disorders, in a rat model of post-infectious cough [95]. In that study, UPLC-Q-TOF-MS/MS identified 94 compounds (33 alkaloids, 22 flavonoids, 9 phenylpropanoids, and 8 triterpene saponins) from KKP. Network pharmacology using the HERB database and SwissTargetPrediction then prioritized candidate targets and pathways, while transcriptomic (RNA-seq) and DIA-based proteomic analyses of lung tissues showed that KKP regulated inflammation- and immunity-related pathways. Functionally, KKP reduced airway and lung pathological injury in a dose-dependent manner (194.4–777.6 mg/kg), decreased levels of inflammatory cytokines (TNF-α, IL-6, IL-1β) in both lung tissue and serum, and alleviated neurogenic inflammation. Importantly, Western blot analysis further confirmed reduced phosphorylation of p65 NF-κB and p38 MAPK—supporting MAPK/NF-κB signaling as a mechanistic pathway involved in the therapeutic effect of KKP. This case provides a clear example of how chemical profiling, network prediction, transcriptomics, proteomics, and targeted molecular validation can be connected into a mechanistic workflow.

Another representative example is Bufei Yishen Formula (BYF) in chronic obstructive pulmonary disease. In this case [113], systems pharmacology was integrated with transcriptomic, proteomic (iTRAQ 8-plex LC-MS/MS), and metabolomic datasets from a cigarette smoke- and *Klebsiella pneumoniae*-induced rat COPD model. Proteomic profiling identified 191 differentially regulated proteins in the COPD model and 195 proteins modulated by BYF treatment, of which 61 shared proteins were reversed by BYF toward normal levels. Instead of relying only on predicted compound–target interactions, the study compared potential BYF targets with regulated transcripts, proteins, and metabolites. The integrated analysis linked BYF treatment to several biological modules, including lipid metabolism (arachidonic acid and linoleic acid metabolism), inflammatory cytokine regulation, antioxidant-related proteins (peroxiredoxin activity), and focal adhesion (13 proteins, *p* = 8.96 × 10^−5^), forming a multi-layer mechanistic picture of BYF action. This example shows how multi-omics integration can connect formula components, molecular targets, metabolic remodeling, and disease phenotypes.

For leukemia and other hematological malignancies, a similar multi-omics strategy represents a logical next step. The molecular landscape of leukemia—characterized by interconnected disruptions in PI3K/Akt, MAPK, and NF-κB signaling, epigenetic dysregulation, and metabolic reprogramming [114]—is well suited to the kind of integrated approach demonstrated above. Network pharmacology could first nominate candidate survival- or inflammation-related pathways; transcriptomic profiling of TCM-treated leukemia models could then determine whether these pathways are transcriptionally remodeled; proteomics could assess corresponding changes in protein abundance and post-translational modifications; and metabolomics could reveal downstream shifts in redox balance, amino acid metabolism, or lipid remodeling. However, as the KKP and BYF cases illustrate, such multi-layer associations remain correlative until they are followed by functional perturbation (e.g., CRISPR-based target knockout) and biochemical binding confirmation. Leukemia, with its well-characterized molecular subtypes, tractable cell line models, and established xenograft systems, provides an ideal disease setting in which to extend the multi-omics-to-mechanism pipeline from respiratory disorders to hematological malignancies.

### 5.4. From Correlation to Causation: Experimental Validation Strategies

The leukemia case, along with the KKP and BYF examples discussed above, exposes the central methodological gap in TCM systems pharmacology. Multi-omics datasets consistently detect coordinated molecular changes, but these observations remain at the level of statistical association. Network models built from such data describe patterns; they do not identify which specific compound–target interactions drive the observed phenotypes. Closing this gap requires a structured validation hierarchy that systematically elevates correlational findings to causal evidence (Figure 5).

#### 5.4.1. Tiered Validation Framework

We propose a four-tier experimental validation framework, ordered by increasing evidentiary strength:

(1) Target prioritization. Computational predictions must first be triaged to focus experimental resources on the most tractable and biologically plausible targets. Prioritization criteria include (i) network topology metrics (degree centrality, betweenness centrality) to identify hub nodes; (ii) druggability assessment using structural databases (e.g., DrugBank, ChEMBL); (iii) availability of validated reagents (antibodies, siRNA, sgRNA design sites); and (iv) prior functional annotation linking the target to the disease phenotype of interest.
(2) Biochemical target engagement. The highest-priority targets should undergo direct binding confirmation. Thermal shift assays (also termed cellular thermal shift assays, CETSA) measure ligand-induced protein thermal stabilization in intact cells. Surface plasmon resonance (SPR) and microscale thermophoresis (MST) provide quantitative binding affinities (Kd) in cell-free systems. Chemoproteomics approaches—including thermal proteome profiling (TPP) and activity-based protein profiling (ABPP)—enable proteome-wide assessment of compound–protein interactions without prior target specification, thereby identifying both on-target and off-target binding events.
(3) Genetic perturbation. Arrayed or pooled CRISPR/Cas9 knockout or CRISPR interference/activation (CRISPRi/a) screens test whether modulating a predicted target alters the pharmacological response. Pooled screens with deep sequencing readouts (e.g., MAGeCK analysis) are suitable for genome-wide target discovery, while arrayed screens allow more detailed phenotypic characterization of prioritized candidates. To resolve cellular heterogeneity, single-cell perturbation sequencing (Perturb-seq) combines CRISPR perturbations with scRNA-seq readout, simultaneously measuring the transcriptional consequences of target modulation in thousands of individual cells.
(4) In vivo functional validation. Target engagement and genetic perturbation evidence from cellular systems must ultimately be tested in disease-relevant animal models. Pathway-specific pharmacological inhibitors, inducible transgenic models, and xenograft assays can determine whether a specific compound–target interaction is necessary and sufficient for the therapeutic effect in vivo.

#### 5.4.2. Bridging Computation and Experiment: An Iterative Loop

The relationship between computational prediction and experimental validation must be bidirectional. Negative experimental results—a predicted target that fails binding assays, or a CRISPR knockout that does not alter the phenotype—provide critical constraints that refine network models. Positive results, in turn, increase confidence in the model and can guide the prediction of additional targets within the same functional module. This iterative feedback loop can gradually improve the accuracy of TCM pharmacology models, transforming descriptive network maps into testable, progressively validated mechanistic models. Practical implementation requires close collaboration between computational and experimental groups, shared data standards (see Section 5.5), and recognition that negative and inconclusive results carry scientific value when they narrow the space of plausible mechanisms.

### 5.5. Standardization, Reproducibility, and FAIR Data Principles

A major source of heterogeneity in TCM multi-omics research is the lack of community-endorsed standards. Addressing this requires action at multiple levels.

Data standards and reporting. Multi-omics studies of TCM should adhere to FAIR (Findable, Accessible, Interoperable, Reusable) data principles. Transcriptomic data should be deposited in GEO or ArrayExpress with complete sample metadata (MIAME/MINSEQE standards). Proteomic data should be submitted to ProteomeXchange via PRIDE, and metabolomic data to MetaboLights, both with minimum reporting guidelines. For network pharmacology, reporting should include compound sources, target prediction tools, database versions, access dates, filtering criteria, and confidence-score thresholds.

Analytical reproducibility. Cross-study reproducibility requires benchmarked integration algorithms, version-controlled analytical pipelines (e.g., Nextflow, Snakemake), and independent replication cohorts. The adoption of containerized workflows (Docker, Singularity) helps analyses be reproduced across computing environments. Inter-laboratory ring trials—in which the same multi-omics dataset is independently analyzed by multiple groups—would identify which analytical choices most strongly influence conclusions and help converge on best practices.

Minimum metadata standards. At minimum, published TCM multi-omics studies should report (i) botanical authentication of herbal materials (voucher specimen numbers); (ii) extraction and preparation methods (solvent, temperature, duration); (iii) administered dose, route, and treatment duration in animal studies; (iv) omics platform specifications (instrument model, software version, database release); and (v) statistical thresholds (FDR correction method, fold-change cutoffs). Community-endorsed checklists, analogous to the MIAME and ARRIVE guidelines, would substantially improve the comparability and cumulative value of published TCM multi-omics research.

## 6. Conclusions and Perspectives

### 6.1. Key Advances

First, high-throughput sequencing and molecular markers have changed how we study geo-authentic medicinal materials, population-level origin traceability using SNPs and InDels, and the epigenetic and metabolic feedback mechanisms that help stabilize those region-specific chemotypes. Second, multi-omics integration has helped us map out the biosynthetic routes for the bioactive compound classes—terpenoids, alkaloids, and flavonoids. The regulatory hierarchy runs from transcription through to chromatin, controlling how much of each pathway gets expressed. Third, network pharmacology and metabolomic profiling have replaced the single-target paradigm with a systems-level view of TCM action. Multi-component formulations engage signaling networks—PI3K/Akt, MAPK, and NF-κB—to produce therapeutic effects. The “genome–biosynthetic pathway–action network” framework captures the process, from resource characterization to mechanistic pharmacology.

### 6.2. The Road Ahead: A Prioritized Action Plan

Translating the multi-omics framework into routine practice requires coordinated action across the TCM research community. We propose the following actionable priorities:

1. Establish community standards and data infrastructure. (i) Adopt minimum metadata standards for TCM multi-omics studies (herbal material authentication, preparation methods, omics platform specifications, statistical parameters). (ii) Deposit raw multi-omics data in public repositories (GEO, ProteomeXchange, MetaboLights) with complete sample metadata as a condition of publication. (iii) Develop curated, species-specific reference databases for medicinal plant genomes, transcriptomes, and metabolomes.
2. Close the causal validation gap. (i) Implement the tiered validation framework outlined in Section 5.4, prioritizing at least one orthogonal validation method (biochemical binding assay, genetic perturbation, or in vivo pathway inhibition) for each computationally predicted mechanism. (ii) Fund collaborative programs linking computational prediction groups with experimental validation laboratories. (iii) Establish shared perturbation screening resources (arrayed CRISPR libraries, chemoproteomics facilities) accessible to TCM research consortia.
3. Bridge biosynthesis and production. (i) Fund coordinated DBTL pilot projects targeting 3–5 high-value TCM natural products, with pre-specified titer, yield, and productivity benchmarks and public reporting of both successes and failures. (ii) Develop standardized techno-economic models that co-optimize strain design, fermentation, and downstream processing. (iii) Address regulatory pathways for fermentation-derived TCM compounds through early engagement with agencies (FDA, EMA, NMPA).

### 6.3. A Closing Note

For a long time, TCM has rested on a holistic premise: therapeutic effects arise from system-level interactions, not from isolated molecular events. Modern systems biology independently arrives at the same conclusion. High-dimensional multi-omics data, interpreted through network models and tested by causal experiments, connect these two traditions. The task is not to reduce TCM to a list of single-target drugs, but to build an evidence-based, mechanistic framework that accommodates the multi-component character of the original formulations. Bringing TCM into precision medicine now turns on the discipline to convert descriptive patterns into testable, causal models of therapeutic action—most of the necessary tools already exist.

## Figures and Tables

**Table 1:** Comparison of DNA barcode markers for medicinal plant authentication.

Marker	Target Genome	Resolution	Limitations
*matK* [20,21]	Plastid	Family–genus level	Low amplification success in some lineages
*rbcL* [20,21]	Plastid	Family–genus level	Low interspecific variation
ITS2 [20,21,24]	Nuclear rDNA	Species level	Primer bias; amplification failure in degraded DNA
psbA-trnH [21]	Plastid	Species level	Length variation complicates alignment
SNP/InDel panels [22]	Nuclear	Population level	Requires prior population genomic data
DNA metabarcoding [23,24]	Multi-locus	Multi-species mixtures	Contamination risk; uneven amplification efficiency

**Table 2:** Comparative summary of representative biosynthetic pathways.

Feature	Artemisinin	Paclitaxel (Taxol)	Flavonoids
Compound class [40,41,42]	Sesquiterpene lactone	Diterpenoid	Polyphenols
Source plant [42,43,44]	*Artemisia annua*	*Taxus* spp.	Ubiquitous
Key committed step [40,41,45]	Amorpha-4,11-diene synthase (ADS)	Taxadiene synthase (TS)	Chalcone synthase (CHS)
Known enzymes [40,42,44]	~10	~19 identified; several mid-pathway steps unresolved	>20 (core pathway well characterized)
Unresolved steps [43,45,46]	Trichome-specific transport	C9 oxidation; C1/C2 hydroxylation; oxetane ring formation	Species-specific tailoring modifications
Key regulators [44,47,48]	AabZIP1, AaGSW1, AaMYC2	JA-responsive TFs (under investigation)	MYB–bHLH–WDR ternary complex
Heterologous production [40,49,50]	Achieved in yeast (artemisinic acid, 25 g/L)	Partial; taxadiene > 1 g/L in *Escherichia coli*	Achieved for many subclasses
Validation strategy [40,44,51]	Enzyme assay + NMR; heterologous reconstitution	Isotopic labeling; heterologous step reconstitution	In vitro enzyme assay; mutant complementation
Most tractable next experiment [45,46,52]	Field-scale semi-synthesis cost reduction	Cryo-EM of multi-enzyme complexes	Engineering tissue-specific glycosylation patterns

**Table 4:** Current omics-based evidence and limitations in TCM-related leukemia studies.

Evidence Type	Intervention	Model	Omics	Samples	Findings	Limitations
Network pharmacology-based studies [1,89]	Various herbal formulas or compounds	AML, CML, and ALL-related models	Network pharmacology, transcriptomic database mining	Variable across studies; sample information is often not consistently reported	Predicted regulation of PI3K/Akt, MAPK, NF-κB, apoptosis, and inflammatory pathways	High dependence on database-based target prediction; high false-positive risk; limited direct target validation
Proteomics-based studies [94]	Single-herb extracts or formula-derived compounds	Leukemia cell-line models	Proteomics, including 2D-DIGE and LC-MS/MS	Mainly cell-line studies; biological replication varies across studies	Differentially abundant proteins are enriched in apoptosis, oxidative stress, redox regulation, and metabolic pathways	Usually lack matched transcriptomic and metabolomic data; limited validation in patient-derived or in vivo models
Transcriptomic studies [112]	TCM-treated hematopoietic or leukemia-related models	Hematopoietic or leukemia-related cells	Bulk RNA-seq	Sample size should be specified according to the original source	Transcriptional reprogramming of apoptosis, immune response, and differentiation-related gene sets	Single-omics evidence; lack of proteomic, metabolomic, biochemical, and functional perturbation validation

## Data Availability

No new data were created or analyzed in this study.
